# A Distribution-Based Metric for Quantifying Dispersibility in Dry Powder Inhalers

**DOI:** 10.3390/pharmaceutics18030283

**Published:** 2026-02-24

**Authors:** Grace Xia, Bhanuz Dechayont, Linze Che, Isabel Comfort, Ashlee D. Brunaugh

**Affiliations:** Department of Pharmaceutical Sciences, College of Pharmacy, University of Michigan, 1007 E. Huron St., Ann Arbor, MI 48104, USA; graxia@umich.edu (G.X.); nuz@umich.edu (B.D.); linzecc@umich.edu (L.C.); icomfort@umich.edu (I.C.)

**Keywords:** pulmonary drug delivery, dry powder inhaler, powder dispersibility, aerosol size distribution, laser diffraction, cascade impaction, Wasserstein distance, optimal transport

## Abstract

**Background/Objectives:** Reproducible evaluation of aerosol dispersibility remains a key challenge in the development of dry powder inhalers (DPIs), where small variations in particle cohesion, morphology, or device resistance can lead to large differences in aerodynamic performance. In passive DPIs, the forces required for powder fluidization and aerosolization arise from the interaction of patient inspiratory airflow with device geometry and must overcome strong interparticle cohesive forces to enable effective lung delivery. Cascade impaction is the gold standard for determining aerodynamic particle size distribution (APSD), but its low throughput and experimental burden limit its utility for systematic formulation and device screening. Prior studies have explored laser diffraction-based particle sizing under varying dispersion energies as indirect metrics of powder dispersibility. Here, we extend this approach by introducing a mathematically rigorous, distribution-based framework that applies the first-order Wasserstein distance (Earth Mover’s Distance) to quantify relative dispersibility with respect to a material-specific maximally dispersed reference state. **Methods:** Mannitol, trehalose, and inulin were spray-dried under matched conditions to generate model dry powders. Particle size distributions were measured by laser diffraction (Sympatec HELOS/R) using both a RODOS dry dispersion module to define a maximally dispersed reference state and an INHALER module to generate aerosols under clinically relevant dispersion conditions spanning multiple device resistances and pressure drops. For each condition, the Wasserstein-1 distance (W_1_) was computed between cumulative volume-based size distributions obtained under reference and inhaler-based dispersion. Cascade impaction was used as an orthogonal method to characterize aerodynamic performance under a representative dispersion condition. **Results**: W_1_ captured formulation-, device-, and flow-dependent differences in dispersibility that were not readily separable by visual inspection of particle size distributions alone. Crystalline mannitol exhibited the largest and most flow-rate-dependent W_1_ values, whereas amorphous trehalose and polymeric inulin showed smaller W_1_ values with distinct, non-monotonic pressure responses that depended on device resistance. W_1_ qualitatively aligned with cascade impaction metrics, exhibiting a positive association with mass median aerodynamic diameter and an inverse association with fine particle fraction, while also demonstrating that efficient dose emission can occur despite incomplete deagglomeration. **Conclusions**: This study establishes the Wasserstein distance as a physically interpretable, formulation-agnostic metric for quantifying aerosol dispersibility relative to a material-specific reference state. This framework enables systematic comparison of dispersion efficiency across devices and operating conditions using standard laser diffraction data and provides a reproducible basis for mechanistic optimization of DPI formulations and inhaler designs.

## 1. Introduction

Dry powder inhalers (DPIs) are widely used for delivering therapies to the respiratory tract, with applications spanning bronchodilators, corticosteroids, antibiotics, and emerging classes such as inhaled biologics and nucleic acid therapeutics. Their appeal lies in device simplicity, portability, chemical stability of solid-state formulations, and the ability to target high local drug concentrations directly to the airways [1,2]. In many cases, active pharmaceutical ingredients (APIs) for delivery via DPI are manufactured as micron-scale particles to reduce the probability of filtration via inertial impaction and enhance deposition in the small airways [3]. However, at this size scale, cohesive and adhesive surface forces dominate over gravitational forces, which promotes particle agglomeration [4,5]. Even when primary particles are initially manufactured within an optimal aerodynamic size range, the extent of fluidization of the powder bed and separation of adhered particles during the inspiratory maneuver ultimately dictates the aerodynamic size distribution governing lung deposition. In passive DPI-based delivery, the shear and flow-induced stresses that promote entrainment and particle interactions arise from the coupling of patient-generated inspiratory flow with inhaler device geometry [6].

Regulatory guidance for orally inhaled drug products reflects this dual nature of dry powder inhaler performance by recommending both geometric particle size characterization of the formulation (e.g., volume-based sizing by laser diffraction) and aerosol performance testing of the delivered dose using cascade impaction under specified flow or pressure drop conditions. These measurements are intended to provide complementary information about the powder as manufactured and the aerosol generated by the device–formulation system, respectively [7]. However, while the guidance recognizes the importance of testing across relevant flow conditions, it does not provide a framework for directly relating a powder’s intrinsic dispersion potential to the extent of dispersion achieved during inhalation. As a result, differences in aerosol performance observed across devices, flow rates, or formulations cannot be readily interpreted in terms of how closely a given inhaler approaches the powder’s dispersion limit, limiting mechanistic insight into deagglomeration efficiency.

Together, these considerations highlight a missing link in current DPI characterization: a quantitative measure of how effectively an inhaler disperses a powder relative to its intrinsic dispersion potential. By comparing inhaler-generated aerosols with those obtained under high-energy, device-independent dispersion conditions that reliably expose the fully dispersed state of the formulation [8], dispersibility can be defined relative to a material-specific reference state rather than inferred solely from aerodynamic outcomes. Distribution-level comparison metrics, such as the Wasserstein (Earth Mover’s) distance, provide a principled and physically interpretable means of quantifying differences between entire particle size distributions [9,10]. Because the Wasserstein distance captures global redistribution across the size spectrum rather than isolated percentiles, it is well suited for problems in which distributional shape carries mechanistic significance [11,12].

Accordingly, the aim of this study is to introduce and experimentally validate a reference-based, distribution-level metric for assessing dry powder dispersibility using the Wasserstein distance. By comparing inhaler-generated particle size distributions with those obtained under high-energy dispersion conditions, this approach quantifies how closely a given inhaler approaches a powder’s intrinsic dispersion limit. We demonstrate that this framework reveals mechanistic differences in deagglomeration across devices and operating conditions, and provides a scalable basis for evaluating DPI powders.

## 2. Materials and Methods

### 2.1. Materials

Trehalose dihydrate (Fisher Scientific, Waltham, MA, USA), mannitol (Thermo Scientific, Waltham, MA, USA), and inulin (TCI Chemicals, Portland, OR, USA) were selected as model excipients to generate dry powder formulations with distinct physicochemical properties. Sulforhodamine B sodium salt (Fisher Scientific, Waltham, MA, USA) was used as a fluorescent tracer to enable quantification of deposited mass during aerodynamic characterization. Distilled and deionized water (MilliQ; MilliporeSigma, Burlington, MA, USA) was used for preparation of all aqueous solutions.

Capsule-based dry powder inhaler devices from the RS01 platform (Amcor, formerly Berry Global, Osango, IT) were used as the model DPI system. Devices with low, medium, and high intrinsic airflow resistances (RS01 Mod 7 variants for size 3 capsules) were employed to evaluate the influence of device resistance on powder dispersion.

### 2.2. Preparation of Model Dry Powder Formulations

Model dry powder formulations were prepared from excipient solutions using a laboratory-scale spray dryer (B-290; BÜCHI Labortechnik AG, Flawil, Switzerland) with operating parameters adapted from previous work by Brunaugh et al. [13]. Feed solutions were prepared at a concentration of 1% (*w*/*v*) in distilled and deionized water and gently stirred until complete dissolution. Sulforhodamine B sodium salt (0.05% *w*/*v*) was included as a fluorescent tracer to enable quantification of deposited mass during aerodynamic characterization.

Atomization was carried out using a two-fluid nozzle equipped with a 0.7 mm nozzle cap and a 1.5 mm nozzle tip. The inlet air temperature was set to 130 °C, resulting in outlet temperatures between 73 and 80 °C. Feed solutions were delivered at approximately 2.0 mL/min using the peristaltic pump. Nitrogen was used as the atomizing gas at a flow rate of 22.9 L/min, and the drying airflow rate was maintained at 35 m^3^/h. To minimize moisture exposure, collected powders were immediately transferred to glass vials under nitrogen and stored at −80 °C until analysis. Prior to characterization, vials were equilibrated from −20 °C to room temperature.

### 2.3. Baseline Characterization of Model Dry Powder Formulations

Baseline characterization was performed to establish the physicochemical properties and intrinsic dispersion limits of the spray-dried model formulations prior to inhaler-based aerosolization. These measurements are not intended as standalone descriptors, but rather to define material-specific reference states against which inhaler-generated aerosols are compared.

#### 2.3.1. Definition and Measurement of the Maximally Dispersed Reference State

Baseline particle size distributions corresponding to the maximally dispersed reference state were measured by laser diffraction using a HELOS/R system (Sympatec GmbH, Clausthal-Zellerfeld, Germany) equipped with the RODOS dry dispersion module and R3 lens. RODOS generates strong, well-controlled aerodynamic stresses through compressed-air acceleration and nozzle impaction. Systematic dry-dispersion titrations show that, as dispersion energy increases, powders undergo a characteristic transition from cohesive agglomerates to progressively smaller units until reaching a plateau where additional energy produces no further size reduction. This plateau behavior, extensively documented by Jaffari et al. [8], provides direct experimental evidence that RODOS achieves a material-specific maximally dispersed state under dry conditions. For some materials, this state corresponds to isolated primary particles, whereas for others it reflects structurally fused units formed during spray drying or storage. In all cases, the plateau represents the smallest physically stable entities present in the powder and therefore defines a practical, material-grounded upper bound on deagglomeration.

To experimentally evaluate whether the selected RODOS operating pressure (3.0 bar) lies within a regime of diminishing dispersion returns, a dispersion-energy titration was conducted for each formulation. Laser diffraction measurements were performed using the RODOS module at dispersion pressures of 0.5, 1.0, 2.0, and 3.0 bar. Single measurements were acquired at 0.5, 1.0, and 2.0 bar to capture the progression of dispersion behavior, while triplicate measurements were performed at 3.0 bar to define the reference distribution used throughout the study. For each formulation, Wasserstein-1 (W_1_) distances were calculated between cumulative particle size distributions measured at each lower dispersion pressure and the pooled, replicate-averaged 3.0 bar reference distribution. This approach quantifies distributional convergence toward the high-energy reference state as dispersion energy increases and provides a distribution-level assessment of whether additional dispersion energy yields substantial redistribution of particle volume across the size axis. Cumulative particle size distributions and corresponding W_1_ distances relative to the 3.0 bar reference are provided in Supplementary Figure S1.

Powders were introduced via a vibratory feeder and dispersed under controlled pressure conditions (3.0 bar), which were selected to reliably achieve the dispersion plateau identified in prior titration studies. Measurements were performed in triplicate for each formulation. Raw diffraction data were processed using Sympatec PAQXOS software (version is 5.1.0) with a built-in Mie Extended Evaluation (MIEE) model to obtain volume-based cumulative particle size distributions.

#### 2.3.2. Scanning Electron Microscopy

Particle morphology of the spray-dried powders in their maximally dispersed state was examined using scanning electron microscopy (SEM) (JSM-7800F LV; JEOL Ltd., Tokyo, Japan). Prior to imaging, powders were dispersed onto double-sided carbon tape using the RODOS dry dispersion system to minimize agglomeration and enable visualization of primary and structurally fused particles. Samples were mounted on 12 mm aluminum stubs and sputter-coated with gold for 90 s under argon using a Denton Desk II sputter coater. SEM imaging was performed under identical preparation and imaging conditions for all formulations.

#### 2.3.3. X-Ray Powder Diffraction

Solid-state properties were assessed by X-ray powder diffraction (XRD) using a Rigaku Miniflex 600 diffractometer (Rigaku Corporation, Tokyo, Japan) equipped with a Cu-Kα radiation source operated at 40 kV and 15 mA. Samples were scanned in continuous mode from 5° to 45° 2θ at a scan rate of 5°/min with a step size of 0.01°.

#### 2.3.4. BET-Specific Surface Area

Specific surface area (SSA) was determined using inverse gas chromatography (IGC-SEA; Surface Measurement Systems, London, UK) with n-octane as a non-polar probe. Powder samples were packed into silanized glass columns and preconditioned under dry nitrogen (10 mL/min, 0% RH) at 30 °C for 2 h prior to analysis. Octane injections were performed under a finite-concentration regime, with fractional surface coverage decreasing stepwise from approximately 0.5 to <0.01 n/nm to construct adsorption isotherms. Methane was used as an inert reference to determine column dead time. BET monolayer capacity and SSA were calculated from the linear region of the BET adsorption isotherm using the BET equation, as implemented in the manufacturer’s software. Only the linear BET region was used for SSA determination.

### 2.4. Aerosol Performance of Model Formulations

Aerosol performance was characterized using both cascade impaction and laser diffraction to capture complementary aspects of inhaler-generated dispersion behavior. Cascade impaction was used to quantify conventional aerodynamic deposition metrics under standardized conditions, while laser diffraction was used to measure volume-based particle size distributions across multiple device resistances and pressure drops.

#### 2.4.1. Laser Diffraction

Inhaler-generated particle size distributions were measured by laser diffraction using a Sympatec HELOS/R system equipped with the INHALER dispersion module. Approximately 20 mg of powder was loaded into size 3 HPMC capsules (Zephyr; Lonza, Basel, Switzerland) and dispersed using RS01 Model 7 dry powder inhalers with low, medium, or high intrinsic airflow resistance.

For each formulation, aerosolization was evaluated across three nominal pressure drops (1, 2, and 4 kPa) for each device resistance. Pressure drops were controlled by adjusting the software-defined depression across the INHALER Venturi tube (24–96 mbar), corresponding to the volumetric flow rates required to achieve the target pressure drop for each device, which were calculated using the manufacturer provided device resistances [6]. Prior to each replicate actuation, a blank measurement was performed using an empty capsule. All measurements were conducted in triplicate for each formulation–device–pressure combination.

Raw diffraction data were processed using Sympatec PAQXOS software with background subtraction and application of the built-in MIEE model. Particle size distributions were exported as cumulative volume-based distributions, Q_3_(x), for subsequent analysis. Volume-based distributions were used to ensure alignment with dose-relevant aerosol metrics, as volume weighting reflects the contribution of each size fraction to the total dispersed mass.

#### 2.4.2. Cascade Impaction

As an orthogonal confirmation of the LD-based APSD measurements, cascade impaction analysis was performed using a Next Generation Impactor (NGI; TSI Incorporated, Shoreview, MN, USA) operated without a pre-separator. Studies were conducted in triplicate for each formulation. To minimize particle bounce, NGI stages were coated with 1% (*v*/*v*) glycerol in ethanol and allowed to dry prior to use. To represent intermediate conditions of those studied using the LD approach, the powders were dispersed using a medium-resistance RS01 DPI at a volumetric flow rate set to achieve a 2 kPa pressure drop across the device (56.6 L/min, based on the reported intrinsic device resistance of 0.025 kPa^1/2·min/L). In accordance with USP <601>, each actuation was performed for a duration sufficient to draw 4 L of air through the apparatus (4.2 s) [14].

Deposited powder was recovered from the inhaler, mouthpiece adaptor, induction port, NGI stages 1–7, and the micro-orifice collector (MOC) by washing with freshly prepared and filtered 0.1 M phosphate-buffered saline (PBS). Dye mass was quantified by UV absorbance at 560 nm using a microplate reader (Epoch2; BioTek Instruments, Winooski, VT, USA). Stage cutoff diameters were calculated using archival NGI stage cut-size equations and coefficients reported by Marple et al. [15].

Aerodynamic deposition parameters were calculated from recovered dye mass. Emitted fraction (EF) was defined as the percentage of total recovered dye emitted from the device. Fine particle fraction (FPF < 5 µm) was calculated as the percentage of emitted dose deposited on NGI stages with aerodynamic cutoff diameters below 5 µm, determined by summing the mass collected on stage 2 (4.60 µm cutoff) through the micro-orifice collector (MOC). Mass median aerodynamic diameter (MMAD) and geometric standard deviation (GSD) were calculated from probit-transformed cumulative mass distributions plotted against logarithmic aerodynamic diameter. Mass balance, defined as the total recovered dye relative to the nominal loaded dose, remained within 85–115% for all experiments.

### 2.5. Calculation of the Wasserstein Distance

To quantify dispersibility as a distribution-level property, the difference between inhaler-generated aerosols and the corresponding maximally dispersed reference state was evaluated using the first-order Wasserstein distance (W_1_). This metric compares entire particle size distributions rather than discrete percentiles or cutpoints, enabling global differences in dispersion behavior to be captured in a single continuous value. Because W_1_ operates on entire distributions rather than replicate-level scalar outputs, it is treated here as a descriptive, physically interpretable effect-size metric rather than a target of inferential statistical testing.

#### 2.5.1. Representation of Particle Size Distributions

All particle size distributions were represented as cumulative volume-based distributions, Q_3_(x), derived from laser diffraction measurements. Cumulative representations were used in place of histogram-based metrics to reduce sensitivity to binning choices and local noise, which can obscure meaningful differences in cohesive powders. This approach is consistent with prior theoretical and empirical analyses of distributional robustness [16,17] and with ISO 13320:2020 recommendations for laser diffraction reporting [18].

For each formulation, the maximally dispersed reference state was defined by the cumulative distribution obtained under high-energy RODOS dispersion conditions. Inhaler-generated aerosols were represented by cumulative distributions measured under each device resistance and pressure drop condition. All distributions were normalized such that Q_3_(∞) = 1, ensuring direct comparability.

#### 2.5.2. Mathematical Formulation of the Distributions

The Wasserstein-1 distance quantifies the minimal redistribution required to transform one cumulative distribution into another. In one dimension, this distance is given by the integral of the absolute difference between the two cumulative distribution functions:

(1) $${W_{1}(F}_{R},\;F_{D})=\int_{0}^{x}\left|F_{R}\left(x\right)-F_{D}\left(x\right)\right|dx$$

Because W_1_ aggregates differences across the entire size axis rather than emphasizing local deviations, it is relatively robust to measurement variability and tail noise, which is advantageous for laser diffraction data [9]. In the context of particle size analysis, W_1_ distance can be understood as a measure of how much particle volume must be “moved” along the particle size axis to transform one distribution into another. When applied to cumulative, volume-based particle size distributions, W_1_ quantifies the total amount of size redistribution required for an inhaler-generated aerosol to reach a defined reference state.

Conceptually, if two distributions differ primarily because one contains a larger fraction of coarse agglomerates, W_1_ reflects the cumulative particle volume that would need to shift from larger to smaller sizes to match the reference distribution. Conversely, if differences are confined to a narrow size range or involve only a small fraction of the total particle volume, W_1_ remains small. In this way, W_1_ provides a physically intuitive, distribution-level measure of dispersibility that accounts for both the magnitude of particle size differences and where those differences occur along the size axis, rather than focusing on a single percentile of the distribution.

#### 2.5.3. Computational Implementation

Wasserstein distances were calculated in R (version 2022.12.0+353). Cumulative volume distributions exported from Sympatec PAQXOS were converted from percent to probability form by scaling *Q_3_(x)* from 0–100% to $F\left(x\right)\in [0,1]$. For each formulation and operating condition, triplicate cumulative distributions were pooled and averaged at each reported particle size increment to generate a single representative inhaler-generated distribution and a single RODOS reference distribution.

The Wasserstein-1 distance was then computed numerically using the discrete particle size grid from the laser diffraction export. Specifically, $W_{1}$ was approximated as a Riemann-sum of the absolute difference between cumulative distributions evaluated at each diameter increment:

$$W_{1}\approx \sum_{i=1}^{n-1}\left|F_{R}\left(x_{i}\right)-F_{D}\left(x_{i}\right)\right|(x_{i+1}-x_{i})$$

where $x_{i}$ denotes the particle diameter grid (µm). Calculations were performed over the common diameter range reported for each paired comparison.

For each formulation, W_1_ values were computed across all device resistance and pressure drop conditions by comparison to the fixed RODOS reference distribution. These values were used to construct flow-dependent dispersibility profiles. Lower W_1_ values indicate that an inhaler-generated aerosol closely approximates the maximally dispersed reference state, reflecting efficient deagglomeration. Higher W_1_ values indicate greater residual cohesion and incomplete dispersion.

### 2.6. Statistical Analysis

Statistical significance of aerosol performance metrics from cascade impaction studies (MMAD, EF, FPF) was assessed using ANOVA (as appropriate) at α = 0.05 followed by Tukey’s post hoc analysis in GraphPad Prism (version 10.6.1). Wasserstein-1 distances were calculated in R (RStudio version 2026.01.0+392; open-source software). Because each Wasserstein-1 value represents a single distribution-to-distribution comparison per condition (INHALER vs. RODOS) after combining replicate laser diffraction runs, inferential statistical testing was not performed for Wasserstein-1 outcomes. Instead, effect magnitudes were evaluated using descriptive summaries (mean and SD across device and pressure drop conditions, range, and coefficient of variation). Figures were generated in R using ggplot2.

#### Bootstrap Analysis of Wasserstein Distance Uncertainty

To quantify uncertainty in Wasserstein-1 (W1) distances arising from finite experimental replication (*n* = 3 laser diffraction measurements per condition), a nonparametric bootstrap resampling analysis was performed independently for each experimental condition defined by formulation, device resistance, and pressure drop (3 × 3 × 3 = 27 conditions). For each condition, replicate cumulative volume-based particle size distributions for the RODOS reference and the corresponding inhaler-generated aerosol were interpolated onto a common particle size grid. The observed W1 value for that condition was computed from pooled distributions obtained by averaging the three RODOS replicate CDFs and the three inhaler replicate CDFs at each particle size, followed by numerical integration of the absolute difference between the pooled CDFs.

Bootstrap resampling was then performed for 2000 iterations per condition. In each iteration, three RODOS replicates and three inhaler replicates were sampled with replacement from the original replicate sets, averaged to form bootstrap-pooled CDFs, and used to compute a bootstrap W1 value. The resulting bootstrap distribution was summarized by its mean, standard deviation, and 95% percentile-based confidence interval; the bootstrap standard deviation was used as a condition-specific estimate of measurement uncertainty, representing the expected variability in W1 under repeated experiments with finite replication.

To contextualize this uncertainty relative to experimentally meaningful dispersion effects, effect-to-noise ratios were computed for formulation, device resistance, and pressure drop in a descriptive manner. For formulation, the effect magnitude was quantified as the standard deviation of the bootstrap-mean W1 values across formulations within each device–pressure combination, averaged across combinations; the corresponding noise level was defined as the mean bootstrap standard deviation within the same combinations, averaged across combinations. For device resistance and pressure drop, effect magnitudes were quantified within each fixed combination of the remaining factors using the across-level range (max − min) of bootstrap-mean W1 values, then averaged across combinations; noise levels were defined as the mean bootstrap standard deviation within the same combinations. Effect-to-noise ratios were computed as the ratio of effect magnitude to noise level for each factor. This analysis was intended to provide an interpretable measure of separability of factor-driven differences relative to measurement variability, without invoking inferential statistical testing.

## 3. Results

### 3.1. Baseline Particle Size, Morphology and Solid-State Structure of Model Powder Formulations

Baseline particle characteristics of the spray-dried formulations were evaluated using scanning electron microscopy (SEM), powder X-ray diffraction (pXRD), and laser diffraction under high-energy dispersion conditions (RODOS). Together, these measurements establish the intrinsic size scale, morphology, and solid-state structure of each formulation prior to inhaler-based aerosolization.

**Figure 1:**
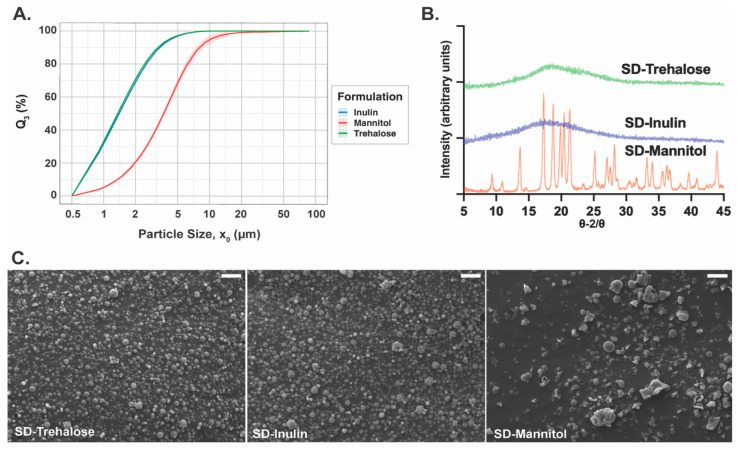
Baseline particle size, solid-state structure, and morphology of spray-dried formulations. (**A**) Cumulative volume particle size distributions (Q_3_) obtained by laser diffraction under RODOS dispersion at 3.0 bar, defining the maximally dispersed reference state for each formulation. Spray-dried inulin and trehalose exhibit similar fully dispersed size profiles, whereas mannitol displays a broader distribution shifted toward larger particle sizes. (**B**) Powder X-ray diffraction (pXRD) patterns of spray-dried trehalose, inulin, and mannitol. Trehalose and inulin show amorphous halo patterns, while mannitol exhibits distinct Bragg reflections consistent with a crystalline structure. (**C**) Scanning electron microscopy (SEM) images of spray-dried particles following dispersion, acquired at identical magnification (2000×) to enable qualitative comparison of primary particle morphology; higher magnification imaging of mannitol was limited by charging artifacts. Trehalose and inulin particles are predominantly spherical, with trehalose appearing smoother and inulin exhibiting greater surface corrugation, whereas mannitol particles display angular, faceted morphologies characteristic of crystalline materials. Scale bars = 5 µm.

Laser diffraction measurements obtained under RODOS dispersion at 3.0 bar were used to define the maximally dispersed reference state for each powder. The resulting cumulative volume distributions (Figure 1A), measured over the detectable size range of the R3 lens (≥0.5 μm), revealed that spray-dried inulin and trehalose exhibited similar fully dispersed size profiles, whereas mannitol displayed a broader distribution shifted toward larger particle sizes. These differences indicate formulation-dependent limits on achievable deagglomeration under high-energy, device-independent dispersion.

Powder X-ray diffraction patterns (Figure 1B) further distinguished the formulations by solid-state structure. Spray dried inulin and trehalose diffractograms exhibited broad halos with no discernible Bragg peaks, reflective of an amorphous nature. In contrast, spray dried mannitol displayed multiple sharp diffraction peaks consistent with a crystalline structure.

SEM imaging of dispersed powders (Figure 1C) revealed formulation-dependent differences in particle morphology. Spray-dried inulin particles exhibited irregular, corrugated surfaces with largely spherical overall geometry, consistent with early vitrification and limited surface relaxation during droplet drying. Trehalose particles were predominantly spherical with comparatively smoother surfaces, indicative of greater molecular mobility and densification prior to vitrification. In contrast, mannitol powders displayed a heterogeneous population consisting of both spherical particles and angular, non-spherical morphologies characteristic of crystalline materials.

BET-specific surface area (BET-SSA) values derived from n-octane adsorption isotherms are summarized in Table 1. Distinct differences in accessible surface area were observed among the three spray-dried formulations. Inulin exhibited the highest BET-SSA (6.99 m^2^/g), followed by trehalose (6.01 m^2^/g), while mannitol showed a substantially lower BET-SSA (2.19 m^2^/g).

Linearized BET fits demonstrated good agreement with the BET model for all formulations, with coefficients of determination (R^2^ ≥ 0.98). For inulin and mannitol, the linear fitting region spanned relative pressures (P/P_0_) from 0.02 to 0.35. For trehalose, the lowest relative pressure points were excluded and fitting was performed over P/P_0_ = 0.05–0.35 to minimize leverage effects at low surface coverage. Across all formulations, the selected fitting ranges provided sufficient data density and linearity for robust estimation of monolayer capacity.

The ranking of BET-SSA values (inulin > trehalose ≫ mannitol) indicates formulation-dependent differences in octane-accessible surface area that are not captured by particle size measurements alone.

### 3.2. Aerosol Performance of Model Formulations as Determined Using Laser Diffraction

**Figure 2:**
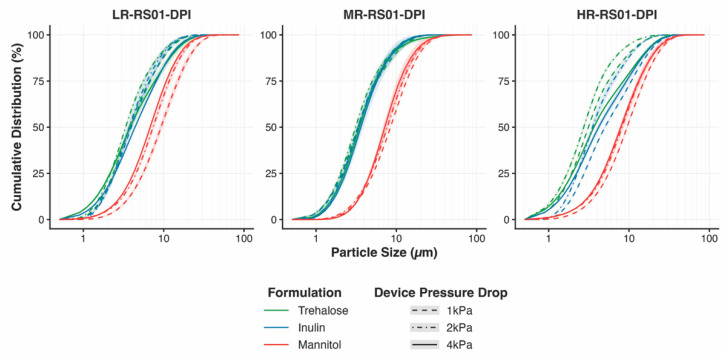
Cumulative volume-based particle size distributions (Q_3_) of inhaler-generated aerosols measured by laser diffraction for spray-dried inulin, mannitol, and trehalose dispersed using RS01 devices spanning low (LR), medium (MR), and high (HR) resistance. For each device, aerosols were generated at pressure drops of 1, 2, and 4 kPa. Distributions are shown by device to highlight resistance-dependent and formulation-specific dispersion behavior. While increasing pressure drop generally promotes finer aerosolization, the magnitude and linearity of this response vary by formulation and device resistance. Medium-resistance devices exhibit comparatively robust dispersion across pressure drops for all formulations, whereas low- and high-resistance devices reveal stronger, formulation-dependent flow-rate effects and increased separation between otherwise similar formulations.

Laser diffraction was used to characterize inhaler-generated aerosol size distributions across devices spanning low, medium, and high resistance under multiple pressure drops (Figure 2). When viewed by device, distinct formulation- and resistance-dependent dispersion behaviors emerged.

In the low-resistance device, mannitol exhibited the greatest sensitivity to inspiratory flow rate. Progressive increases in pressure drop from 1 to 4 kPa produced pronounced leftward shifts in the cumulative size distribution, indicating substantial flow-rate–dependent deagglomeration. This flow-rate dependence was attenuated in the medium-resistance device and was less consistently observed under high-resistance conditions.

For trehalose and inulin, changes in the emitted particle size distribution as a function of inspiratory flow rate were minimal in the medium-resistance device relative to both the low- and high-resistance devices, indicating greater robustness to flow-rate variability under intermediate resistance. In contrast, under low- and high-resistance conditions, both formulations exhibited non-linear flow-rate dependence, with the 4 kPa condition producing rightward shifts in the cumulative distribution relative to 2 kPa. This effect was most pronounced for the high-resistance device.

Notably, under high-resistance conditions, trehalose and inulin also exhibited increased separation in their cumulative size distributions at a given pressure drop, despite their baseline similarities in physicochemical properties and their comparable performance in the medium-resistance device. This divergence indicates that inhaler resistance can amplify formulation-specific dispersion behavior that is not evident from intrinsic particle characteristics alone.

### 3.3. Orthogonal Cascade Impaction Studies

Cascade impaction was used as an orthogonal measurement to the laser diffraction–based aerosol performance assessment, using the medium-resistance RS01 DPI operated at a 2 kPa pressure drop. This condition corresponds to the midpoint of the dispersion space explored by laser diffraction and was selected as a representative benchmark.

**Figure 3:**
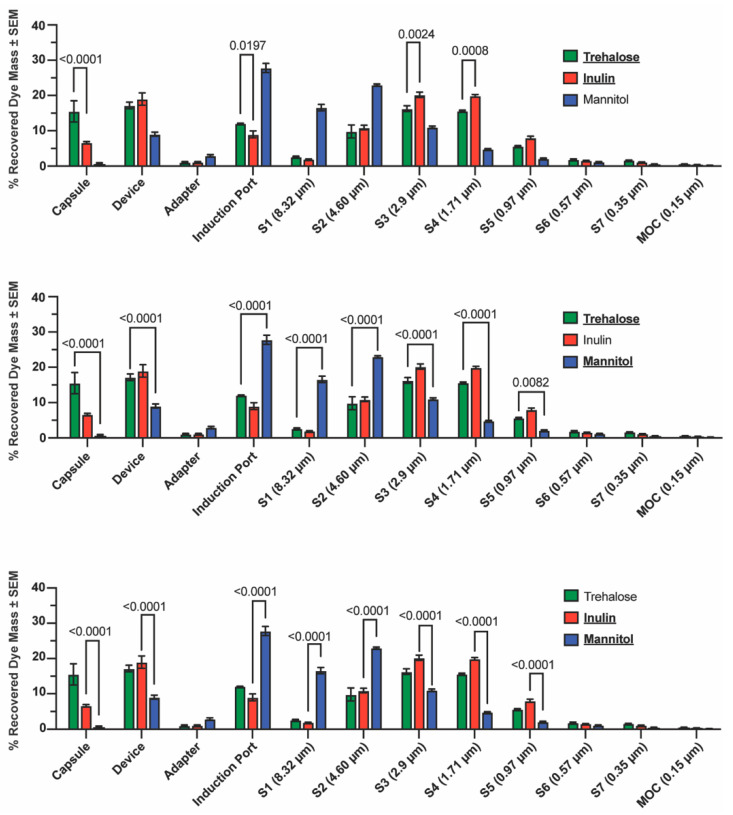
Cascade impaction-derived aerodynamic particle size distributions for spray-dried trehalose, inulin, and mannitol aerosols generated using the medium-resistance RS01 DPI operated at a 2 kPa pressure drop. Bars represent the percentage of recovered Sulforhodamine B mass deposited in each NGI component and stage (mean ± SEM, *n* = 3). Statistical analysis was performed using two-way ANOVA with formulation and collection stage as factors, followed by Tukey’s multiple comparisons test. For clarity, individual panels display selected pairwise post hoc comparisons highlighting statistically significant differences between formulations at specific collection stages; the underlying dataset and statistical analysis are identical across panels. Significant differences (*p* < 0.05) are indicated above brackets. Stage cutoff diameters (D_50_, 50% collection efficiency) are shown in parentheses.

Mass deposited in NGI stages 1–5 differed significantly between the spray-dried mannitol formulation and both spray-dried inulin and trehalose formulations, consistent with the pronounced rightward shift observed in the mannitol cumulative size distribution under the same dispersion conditions by laser diffraction (Figure 3). Spray-dried inulin exhibited significantly greater deposition in NGI stages 3 and 4 compared with spray-dried trehalose, indicating a finer emitted aerosol under these conditions.

These formulation-dependent differences were reflected in the mass median aerodynamic diameter (MMAD) values derived from the cascade impaction data (Table 2). Spray-dried mannitol exhibited a substantially larger MMAD (6.36 ± 0.33 µm) relative to both spray-dried trehalose (2.68 ± 0.19 µm) and spray-dried inulin (2.64 ± 0.06 µm), consistent with its broader and coarser aerosol size distribution. In contrast, trehalose and inulin formulations produced comparably fine aerosols by aerodynamic classification, with closely aligned MMAD values despite modest differences in stage-level mass deposition.

Notably, the relative ordering of formulations by MMAD closely mirrored the ordering of median particle sizes (X50) obtained from inhaler-based laser diffraction under matched dispersion conditions, with mannitol producing the largest central tendency values and trehalose and inulin producing smaller values. While absolute MMAD and X50 values differed, reflecting the distinct physical principles underlying inertial impaction and optical ensemble sizing, the preservation of formulation-dependent trends across both techniques indicates that differences in central tendency of the aerosol particle size distribution are robust to the measurement modality.

Together, these results demonstrate that formulation-dependent differences in aerosol size distributions observed by laser diffraction are preserved under aerodynamic classification by cascade impaction, supporting the use of laser diffraction-derived distributions as a structurally meaningful basis for dispersibility analysis.

### 3.4. Quantification of Formulation-, Device-, and Flow-Dependent Dispersion Using Wasserstein-1 Distance

While laser diffraction cumulative size distributions revealed clear formulation- and device-dependent trends, visual comparison alone did not allow clean separation of pressure and device effects across all formulations. To quantitatively capture these multidimensional dispersion behaviors, the one-dimensional Wasserstein distance (W_1_) was calculated between inhaler-generated aerosol size distributions and the corresponding maximally dispersed RODOS reference for each formulation.

**Figure 4:**
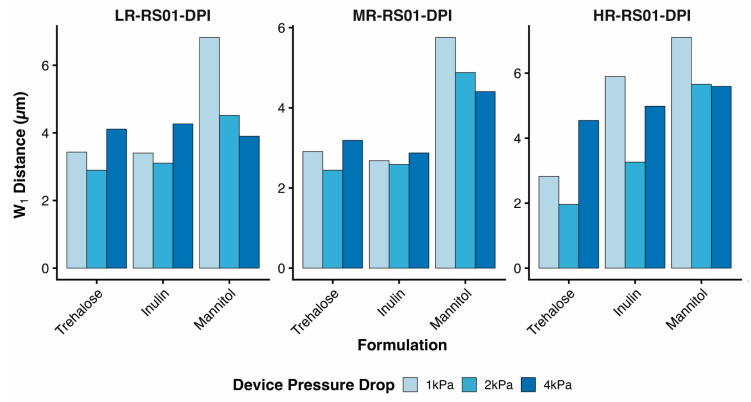
Quantification of formulation- and device-dependent dispersion behavior using the Wasserstein-1 (W_1_) distance. W_1_ distances were calculated between inhaler-generated cumulative particle size distributions and the corresponding maximally dispersed RODOS reference state for trehalose, inulin, and mannitol across low-, medium-, and high-resistance RS01 DPI devices operated at pressure drops of 1, 2, and 4 kPa. Bars represent the integrated distributional deviation (µm) between inhaler and reference conditions, with larger W_1_ values indicating greater departure from the fully dispersed state. Data are grouped by formulation and faceted by device resistance to highlight formulation-specific sensitivity to device configuration and pressure drop.

Figure 4 presents W_1_ distances as a function of formulation and pressure drop, faceted by device resistance. This representation compresses the full cumulative size distribution information into a single physically interpretable metric while preserving the interaction structure observed in the raw distributions.

Bootstrap resampling revealed that W_1_ values exhibited low within-condition variability across the experimental design space. The mean bootstrap-derived standard deviation across all formulation–device–pressure combinations was 0.198 µm, indicating that repeated execution of the same experimental condition would be expected to yield W_1_ values within approximately ±0.2 µm due to measurement uncertainty alone. In contrast, variation in W_1_ associated with changes in formulation, device resistance, and pressure drop substantially exceeded this measurement noise. The formulation effect magnitude, quantified as the standard deviation of mean W_1_ values across formulations within fixed device–pressure combinations, was 1.41 µm. Device resistance and pressure drop effects, quantified as the across-level range (max − min) of mean W_1_ values within fixed combinations of the remaining factors, were 0.82 µm and 0.90 µm, respectively. These correspond to effect-to-noise ratios of 7.1 for formulation, 4.1 for device resistance, and 4.5 for pressure drop. Together, these results indicate that all three experimental factors produce W_1_ changes several-fold larger than intrinsic measurement uncertainty, supporting the robustness and interpretability of the observed dispersibility trends (Supplementary Figure S2).

Across all device configurations, mannitol exhibited substantially larger W_1_ distances than both inulin and trehalose, consistent with its broader, right-shifted cumulative size distributions observed by laser diffraction. In the low-resistance device, mannitol showed the strongest pressure dependence, with W_1_ decreasing markedly as pressure drop increased from 1 to 4 kPa, reflecting progressive deagglomeration with increasing dispersion energy. This pressure sensitivity was attenuated in the medium-resistance device and further reduced in the high-resistance device configuration.

In contrast, inulin and trehalose displayed comparatively smaller W_1_ distances across all devices, indicating more limited deviation from their respective RODOS reference states. For these formulations, the medium-resistance device produced the most pressure-robust behavior, with minimal changes in W_1_ across the 1–4 kPa range. In the high-resistance device, however, inulin and trehalose diverged more strongly from one another, despite their similar baseline physicochemical properties and similar maximally dispersed size distributions. Notably, W_1_ did not decrease monotonically with increasing pressure drop for either formulation in this configuration, consistent with the non-linear flow-rate dependence observed in the cumulative size distributions.

Together, these results demonstrate that the Wasserstein distance provides a compact, quantitative measure of aerosol dispersion that preserves formulation-specific and device-specific trends observed in laser diffraction data. By integrating information across the entire size distribution, W_1_ enables direct comparison of dispersion performance across devices and operating conditions that are not readily separable by visual inspection of cumulative distributions alone.

### 3.5. Alignment of Wasserstein-Based Dispersibility with Aerodynamic Cascade Impaction Metrics

**Figure 5:**
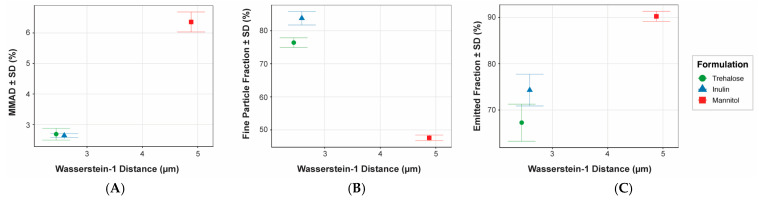
Relationship between Wasserstein-1 (W_1_) distance derived from inhaler-based laser diffraction and aerodynamic cascade impaction metrics obtained under matched dispersion conditions (medium-resistance RS01 DPI, 2 kPa pressure drop). Shown are formulation-level comparisons of (**A**) mass median aerodynamic diameter (MMAD), (**B**) fine particle fraction (FPF < 5 µm), and (**C**) emitted fraction (EF), each plotted as a function of the corresponding W_1_ distance. Data points represent mean ± standard deviation (*n* = 3). Across all metrics, formulations with larger W_1_ distances—indicating greater deviation from the maximally dispersed reference state—exhibited coarser aerodynamic size characteristics and reduced fine particle delivery. The preservation of formulation-dependent ordering across optical ensemble sizing and inertial classification supports the interpretation of W_1_ as a structurally meaningful descriptor of aerosol dispersibility.

To assess whether the Wasserstein-1 distance derived from laser diffraction captures aerosol characteristics relevant to aerodynamic performance, formulation-level W_1_ values were compared with key cascade impaction metrics obtained under matched dispersion conditions (Figure 5). Although the number of formulations precluded formal inferential correlation analysis, clear monotonic relationships were observed across measurement modalities.

Formulations exhibiting larger W_1_ values, indicative of greater deviation from the maximally dispersed reference state, produced aerosols with larger MMADs and reduced fine particle fractions. Mannitol, which showed the largest W_1_ distance by laser diffraction, also exhibited the largest MMAD and lowest FPF by cascade impaction. In contrast, trehalose and inulin clustered closely in both W_1_ and aerodynamic size metrics, consistent with their similar inhaler-generated cumulative size distributions.

Notably, the relative ordering of formulations by W_1_ distance was preserved across all aerodynamic endpoints examined, despite the distinct physical principles underlying optical ensemble sizing and inertial classification. This cross-method consistency supports the interpretation of the Wasserstein distance as a structurally meaningful measure of aerosol dispersibility rather than a device- or method-specific artifact.

While emitted fraction showed an opposing trend, increasing with W_1_ distance, this behavior reflects differences in powder emptying and inertial transport rather than dispersion efficiency per se. Together, these results indicate that W_1_ captures formulation-dependent differences in the size structure of the emitted aerosol, which align with, but are not redundant with, traditional cascade impaction metrics. A consolidated summary of formulation physicochemical properties, reference and inhaler particle size metrics, Wasserstein distances, and aerodynamic performance parameters under matched conditions is provided in Supplementary Table S1 to facilitate cross-comparison across characterization modalities.

## 4. Discussion

Aerosol performance of dry powder inhalers is commonly summarized using scalar descriptors derived from particle size distributions, including mass median aerodynamic diameter (MMAD), median or percentile diameters (e.g., X_50_), fine particle fraction (FPF), and emitted dose (ED). These metrics are well established and play a central role in both formulation development and regulatory evaluation. However, by construction, they reduce the full particle size distribution, which is a high-dimensional object, to a small number of point estimates.

Such reductions are not fully aligned with the underlying physics of aerosol dispersion. Dispersion of cohesive powders does not occur through uniform contraction of the particle size distribution toward smaller diameters; instead, increasing dispersion energy drives complex and often non-monotonic redistribution across the size spectrum, including partial breakup of agglomerates, emergence of fine particle populations, and persistence of larger fragments resistant to further dispersion [19,20,21]. Similar non-linear redistribution behavior has been reported for DPI aerosols consisting of mixtures of primary particles and small agglomerates, for which scalar cascade impaction metrics were shown to inadequately reflect the underlying dispersion process [22].

Cascade impactors do not discretely sort particles into sharp size bins but instead operate through stage-specific collection efficiency curves defined by nominal 50% cut-off aerodynamic diameters [23]. Particles above and below each cut-off deposit with non-zero probability, such that the mass collected on a given stage represents an integral over a range of aerodynamic sizes rather than a point value. The resulting stage deposition profile therefore reflects a convolution of the true aerosol size distribution with overlapping efficiency functions governed by impactor geometry and operating conditions. De Boer et al. demonstrated that under such conditions, particularly for non-log-normal or multimodal aerosols, derivation of a unique MMAD is highly uncertain, with plausible MMAD values inferred from the same impactor dataset differing by more than 1 µm depending on fitting assumptions [24]. The mass median aerodynamic diameter derived from cascade impaction is therefore not directly measured but inferred through regression of probit-transformed cumulative mass fractions, typically under the assumption of a log-normal distribution [14]. As such, MMAD represents a model-dependent central tendency that integrates both dispersion behavior and aerodynamic classification, rather than a direct descriptor of the underlying particle size redistribution process.

A key conceptual advance of the present work is the explicit treatment of deagglomeration as a redistribution process rather than a point shift. The Wasserstein-1 distance provides a mechanistically aligned framework for quantifying aerosol performance by directly measuring how much particle mass must be redistributed, and over what distance in size space, for an inhaler-generated aerosol to match its maximally dispersed reference state [25]. By operating on the entire distribution rather than isolated cutpoints, W_1_ captures differences in distributional shape and dispersion efficiency within a single, physically interpretable metric.

Building on this framework, the present results demonstrate how a reference-based, distribution-level metric reveals dispersion behavior that is not readily separable by visual inspection of particle size distributions alone. Across the formulations studied, the Wasserstein distance captured clear formulation-, device-, and flow-dependent differences in dispersibility that were consistent with material properties yet strongly modulated by inhaler resistance. Crystalline mannitol exhibited the largest W_1_ values and the strongest pressure dependence, particularly in the low-resistance device, indicating a greater redistribution distance required to approach its maximally dispersed reference state. In contrast, amorphous trehalose and polymeric inulin exhibited smaller W_1_ values overall, with dispersion that was relatively insensitive to pressure drop in the medium-resistance device but increasingly device-dependent under high-resistance conditions.

Importantly, these distinctions emerged despite trehalose and inulin exhibiting similar maximally dispersed reference distributions under high-energy RODOS dispersion. By normalizing inhaler performance to a formulation-specific upper bound, the Wasserstein-based approach isolates how efficiently a given device–flow condition accesses a powder’s intrinsic dispersion potential, rather than conflating formulation differences with absolute particle size. As a result, powders with similar baseline size and morphology can be differentiated based on their response to changes in device resistance and dispersion energy, even when scalar descriptors such as median diameters overlap. This distinction reflects the fact that W_1_ probes dispersion efficiency at the point of emission, whereas cascade impaction captures additional downstream aerodynamic classification.

Comparison with cascade impaction further supports the physical relevance of the distribution-based metric while clarifying the distinct information captured by each technique. Under the matched medium-resistance, 2 kPa condition, formulation-dependent ordering by W_1_ was preserved in the corresponding MMAD values, with mannitol producing substantially larger aerodynamic sizes than trehalose and inulin. This qualitative agreement indicates that redistribution distance quantified by W_1_ reflects dispersion behavior that is consequential for aerodynamic classification, even though absolute MMAD and laser diffraction X_50_ values differ due to the fundamentally different measurement principles underlying inertial impaction and optical ensemble sizing.

Notably, trehalose and inulin exhibited similar W_1_ values under this condition despite statistically significant differences in stage-level mass deposition by cascade impaction. This apparent discrepancy reflects the fundamental distinction between pre-aerodynamic dispersion efficiency and post-emission aerodynamic classification. While W_1_ quantifies how efficiently a device disperses a powder relative to its intrinsic dispersion limit at the point of emission, cascade impaction integrates subsequent aerodynamic transport, impaction, and size-dependent inertial sorting into the measured outcome. One plausible explanation is that the observed differences arise from near-field aerodynamic sampling and impaction effects downstream of powder emission. The induction port geometries used in the NGI and laser diffraction systems differ in material, angle, and surface characteristics, and this region acts as a strong classifier immediately following aerosol generation. As a result, subtle formulation-dependent differences in particle inertia or agglomerate structure may be amplified during aerodynamic transport and impaction, even when emitted size distributions measured optically appear similar. This distinction underscores the integrated aerodynamic outcome of cascade impaction, whereas the Wasserstein-based approach isolates dispersion efficiency relative to a material-specific reference state prior to aerodynamic classification.

Under fixed device resistance and operating conditions, the observed differences in dispersibility can be traced to intrinsic material responses to applied dispersion energy. These responses are governed by the combined influence of solid-state form, accessible surface area, and particle morphology, which together determine the nature and strength of interparticle contacts formed during powder consolidation and dispersion-driven collisions.

Crystalline mannitol, characterized by angular, faceted morphology and low BET-specific surface area, forms rigid, discrete contact points that resist deformation. As a result, applied dispersion energy is expended primarily on progressive breakup of persistent coarse fragments, producing a largely monotonic decrease in W_1_ with increasing pressure drop. In contrast, amorphous trehalose and inulin exhibit higher accessible surface areas and deformable particle matrices, enabling larger real contact areas and more heterogeneous agglomerate strength distributions. The rougher, corrugated morphology of inulin promotes a broad distribution of weak contacts, leading to gradual, erosion-like redistribution of particle volume across the size spectrum. Trehalose, which forms smoother and more spherical particles, exhibits fewer but stronger interparticle contacts, resulting in more threshold-dominated deagglomeration in which fine particles are generated while coarse fragments persist. Such distinct breakup pathways may explain why trehalose and inulin can exhibit similar W_1_ values under certain conditions yet diverge in stage-resolved aerodynamic deposition, as subtle differences in agglomerate structure are amplified during downstream aerodynamic classification.

Taken together, these results demonstrate that the Wasserstein distance complements, rather than replaces, established aerodynamic metrics. By decoupling dispersion efficiency from downstream aerodynamic filtering, the proposed framework provides a scalable and device-agnostic means of comparing how closely different inhaler configurations approach a powder’s intrinsic dispersion limit. Because the reference state is defined experimentally for each formulation, this approach is readily extendable to other powders, devices, and operating conditions using standard laser diffraction measurements, and is best interpreted as a mechanistic and early-stage screening tool that contextualizes, rather than supplants, pharmacopeial aerosol performance endpoints.

The present study focuses on spray-dried, single-component powders for which dispersion proceeds primarily through deagglomeration of cohesive particle clusters. In contrast, carrier-based DPI formulations, such as lactose blends, involve fundamentally different dispersion mechanisms dominated by detachment of API particles from carrier surfaces rather than agglomerate breakup. In these systems, dispersion behavior is governed by additional factors including carrier surface roughness, API–carrier adhesion, and redistribution of fines. Consequently, definition of a “maximally dispersed” reference state and interpretation of W_1_ would require formulation-specific adaptation, as a single high-energy dispersion condition may not correspond to complete API liberation. While the reference-based W_1_ framework remains conceptually applicable, its extension to carrier-based systems would require careful selection and validation of an appropriate reference state and is therefore beyond the scope of the present study.

One methodological consideration in the present study is the use of the R3 laser-diffraction lens, which resolves particle diameters down to approximately 0.5 µm. As a result, volume fractions below this threshold are not directly captured, and very fine particle populations, such as those observed in our inulin and trehalose model powders, may be partially truncated. Because the Wasserstein-1 distance integrates redistribution across the full-size axis, unresolved fines could, in principle, contribute to the calculated transport distance.

To assess the potential impact of this limitation, we estimated an upper bound on the contribution of the unresolved sub–0.5 µm size range to the overall W_1_ value using conservative assumptions. Even under a worst-case scenario in which the maximum observed difference in cumulative fines between RODOS and inhaler-generated aerosols persisted uniformly across the entire unresolved interval (0.25–0.5 µm), the resulting incremental contribution to W_1_ was estimated to be on the order of 0.06 µm. Relative to the measured W_1_ values (≈2–3 µm), this corresponds to a maximum fractional error of approximately 2%.

Importantly, the dominant contributions to W_1_ in the present dataset arise from redistribution in the 1–20 µm size range, where the R3 lens provides reliable resolution and where the largest formulation-, device-, and flow-dependent differences were observed. As such, truncation of the extreme fines tail does not materially alter the qualitative trends or mechanistic conclusions drawn from the dispersibility analysis. While use of a lower-cutoff lens (e.g., R2) could further refine quantification of submicron redistribution, the incremental benefit is expected to be small relative to the magnitude of the observed W_1_ differences. Future studies extending this framework to highly respirable or nanoparticle-containing formulations may nonetheless benefit from expanded submicron resolution.

While the reference-based Wasserstein framework enables resolution of intrinsic dispersibility behavior across formulations and device resistances, the present study limits aerodynamic validation to a single, representative operating condition. Because aerodynamic measurements inherently integrate dispersion, transport, and flow-rate-dependent classification effects, extending such measurements across multiple conditions would confound isolation of dispersion-driven behavior. As such, the current work does not claim universal alignment between W_1_ and pharmacopeial aerodynamic metrics across all operating regimes. Broader validation across devices, flow rates, and formulation classes will be required to fully delineate the boundaries of applicability of the W_1_ framework.

Beyond its utility as a descriptive metric, the reference-based Wasserstein framework has practical implications for formulation screening and device–formulation matching. By quantifying how efficiently a given inhaler configuration accesses a powder’s intrinsic dispersion potential, W_1_ enables early differentiation between device-limited and formulation-limited dispersion regimes. In this context, similar aerodynamic outcomes may arise from fundamentally different dispersion pathways, which can be resolved through distribution-level analysis. Accordingly, W_1_ is intended as a mechanistic and comparative dispersibility descriptor to support formulation and device screening, rather than as a standalone regulatory performance metric. Although this study establishes the Wasserstein distance as a mechanistically informative dispersibility metric for single-component DPI powders, extension to more complex formulations and device platforms will require additional validation to define the scope and boundaries of its applicability. Applied during early development, this approach may help identify formulations that are robust to patient flow variability, or devices that inefficiently utilize available dispersion energy, prior to resource-intensive cascade impaction studies.

## Figures and Tables

**Table 1:** BET-SSA values from n-Octane isotherms of spray-dried powders.

Formulation	BET-SSA (m^2^/g)	BET-SSA Fitting Range (P/P_0_)	BET Adsorption Isotherm R^2^
SD-Trehalose	6.01	0.05–0.35 ^1^	0.98
SD-Inulin	6.99	0.02–0.35	0.99
SD-Mannitol	2.19	0.02–0.35	0.99

^1^ Eight data points fitted to construct linearized adsorption isotherm.

**Table 2:** Central tendency of aerosol particle size distributions determined by cascade impaction and inhaler-based laser diffraction (medium-resistance RS01 DPI, 2 kPa).

Formulation	MMAD ± SD (*n* = 3; µm)	X_50_ ± SD (*n* = 3; µm)
SD-Trehalose	2.68 ± 0.19	3.11 ± 0.09
SD-Inulin	2.64 ± 0.06	3.35 ± 0.13
SD-Mannitol	6.36 ± 0.33	7.73 ± 0.30

## Data Availability

The software used to compute Wasserstein-1 (W_1_) distances and perform dispersibility analyses is publicly available at GitHub (https://github.com/Brunaugh-Lab/dispersibility-analysis (accessed on 3 February 2026)) and archived on Zenodo (https://doi.org/10.5281/zenodo.18475101 (accessed on 3 February 2026)). The raw laser diffraction datasets supporting the findings of this study are available via the University of Michigan’s Deep Blue Data repository at https://doi.org/10.7302/nwc8-9f06 (accessed on 15 February 2026) [26].

## Supplementary Materials

The following supporting information can be downloaded at: https://www.mdpi.com/article/10.3390/pharmaceutics18030283/s1, Figure S1: Determination of the maximally dispersed reference state by dry dispersion energy titration using the RODOS module; Figure S2: Bootstrap-based analysis of measurement uncertainty and effect magnitude for Wasserstein-1 (W_1_) distances; Table S1: Comprehensive summary of material properties, morphology, particle size distributions, dispersibility, and aerodynamic performance for spray-dried formulations.

## Abbreviations

The following abbreviations are used in this manuscript:

APSD	Aerodynamic Particle Size Distribution
API	Active Pharmaceutical Ingredient
BET	Brunauer–Emmett–Teller
BET-SSA	Brunauer–Emmett–Teller Specific Surface Area
DPI	Dry Powder Inhaler
ED	Emitted Dose
EF	Emitted Fraction
FDA	Food and Drug Administration
FPF	Fine Particle Fraction
GSD	Geometric Standard Deviation
HELOS/R	Helium-Neon Laser Optical System/R-Series
HR	High Resistance
IGC	Inverse Gas Chromatography
IGC-SEA	Inverse Gas Chromatography-Surface Energy Analyzer
INHALER	Sympatec INHALER Dispersion Module
ISO	International Organization for Standardization
LD	Laser Diffraction
LR	Low Resistance
MIEE	Mie Extended Evaluation
MMAD	Mass Median Aerodynamic Diameter
MOC	Micro-Orifice Collector
MR	Medium Resistance
NGI	Next Generation Impactor
PBS	Phosphate-Buffered Saline
pXRD	Powder X-ray Diffraction
Q_3_(x)	Cumulative Volume-Based Particle Size Distribution
RF	Respirable Fraction
RODOS	RODOS Dry Dispersion Unit
RS01	Capsule-Based Dry Powder Inhaler Platform
SEM	Scanning Electron Microscopy
SSA	Specific Surface Area
USP	United States Pharmacopeia
UV-Vis	Ultraviolet-Visible
W_1_	First-Order Wasserstein Distance
X_50_	Median Particle Diameter

## References

[1] de Boer A.H., Hagedoorn P., Hoppentocht M., Buttini F., Grasmeijer F., Frijlink H.W. (2017). Dry powder inhalation: Past, present and future. Expert Opin. Drug Deliv..

[2] Brunaugh A.D., Smyth H.D.C. (2018). Formulation techniques for high dose dry powders. Int. J. Pharm..

[3] Patton J.S., Byron P.R. (2007). Inhaling medicines: Delivering drugs to the body through the lungs. Nat. Rev. Drug Discov..

[4] Hickey A.J., Mansour H.M., Telko M.J., Xu Z., Smyth H.D.C., Mulder T., McLean R., Langridge J., Papadopoulos D. (2007). Physical Characterization of Component Particles Included in Dry Powder Inhalers. I. Strategy Review and Static Characteristics. J. Pharm. Sci..

[5] Visser J. (1989). Van der Waals and other cohesive forces affecting powder fluidization. Powder Technol..

[6] Clark A.R., Weers J.G., Dhand R. (2020). The Confusing World of Dry Powder Inhalers: It Is All About Inspiratory Pressures, Not Inspiratory Flow Rates. J. Aerosol. Med. Pulm. Drug Deliv..

[7] U.S. Food and Drug Administration (2018). Metered Dose Inhaler (MDI) and Dry Powder Inhaler (DPI) Drug Products—Quality Considerations.

[8] Jaffari S., Forbes B., Collins E., Barlow D.J., Martin G.P., Murnane D. (2013). Rapid characterisation of the inherent dispersibility of respirable powders using dry dispersion laser diffraction. Int. J. Pharm..

[9] Peyré G., Cuturi M. (2020). Computational Optimal Transport. arXiv.

[10] Flamary R., Courty N., Gramfort A., Alaya M.Z., Boisbunon A., Chambon S., Chapel L., Corenflos A., Fatras K., Fournier N. (2021). POT: Python Optimal Transport. J. Mach. Learn. Res..

[11] Garrett R., Harris T., Wang Z., Li B. (2024). Validating climate models with spherical convolutional Wasserstein distance. Adv. Neural Inf. Process. Syst..

[12] Rubner Y., Tomasi C., Guibas L.J. (2000). The Earth Mover’s Distance as a Metric for Image Retrieval. Int. J. Comput. Vis..

[13] Brunaugh A.D., Wu T., Kanapuram S.R., Smyth H.D. (2019). Effect of particle formation process on characteristics and aerosol performance of respirable protein powders. Mol. Pharm..

[14] United States Pharmacopeial Convention (2025). <601> Inhalation and Nasal Drug Products: Aerosols, Sprays, and Powders—Performance Quality Tests. United States Pharmacopeia and National Formulary (USP–NF).

[15] Marple V.A., Olson B.A., Santhanakrishnan K., Mitchell J.P., Murray S.C., Hudson-Curtis B.L. (2003). Next generation pharmaceutical impactor (a new impactor for pharmaceutical inhaler testing). Part II: Archival calibration. J. Aerosol. Med..

[16] Park S.R., Kolouri S., Kundu S., Rohde G.K. (2018). The cumulative distribution transform and linear pattern classification. Appl. Comput. Harmon. Anal..

[17] Schlunk S., Byram B.C. (2023). Methods for Enhancing the Robustness of the Generalized Contrast-to-Noise Ratio. IEEE Trans. Ultrason. Ferroelectr. Freq. Control.

[18] (2020). Particle Size Analysis—Laser Diffraction Methods.

[19] Tong Z., Yu A., Chan H.-K., Yang R. (2015). Discrete Modelling of Powder Dispersion in Dry Powder Inhalers—A Brief Review. Curr. Pharm. Des..

[20] Wong W., Fletcher D.F., Traini D., Chan H.-K., Crapper J., Young P.M. (2010). Particle Aerosolisation and Break-up in Dry Powder Inhalers 1: Evaluation and Modelling of Venturi Effects for Agglomerated Systems. Pharm. Res..

[21] Behara S.R.B., Larson I., Kippax P., Morton D.A.V., Stewart P. (2011). The kinetics of cohesive powder de-agglomeration from three inhaler devices. Int. J. Pharm..

[22] Grasmeijer F., Hagedoorn P., Frijlink H.W., de Boer A.H. (2012). Characterisation of high dose aerosols from dry powder inhalers. Int. J. Pharm..

[23] Marple V.A. (2004). History of Impactors—The First 110 Years. Aerosol. Sci. Technol..

[24] de Boer A.H., Gjaltema D., Hagedoorn P., Frijlink H.W. (2002). Characterization of inhalation aerosols: A critical evaluation of cascade impactor analysis and laser diffraction technique. Int. J. Pharm..

[25] Villani C. (2009). The Wasserstein distances. Optimal Transport: Old and New.

[26] Brunaugh A., Xia G. Raw Laser Diffraction Aerosol Size Distribution Data Supporting Wasserstein-Based Dispersibility Analysis of Dry Powder Inhalers [Data Set], University of Michigan—Deep Blue Data. https://deepblue.lib.umich.edu/data/anonymous_link/show/66dd758ce396103d19a0b3104b1a7a24bc2f65aa9f7cca6b654cc80a9541fd6e?locale=en.

