# Supplementary material for: A Distribution-Based Metric for Quantifying Dispersibility in Dry Powder Inhalers
Source: Pharmaceutics. 2026 Feb 24;18(3):283. doi: 10.3390/pharmaceutics18030283 (PMC13028960; doi:10.3390/pharmaceutics18030283)
Supplement: Supplementary file 1 [file pharmaceutics-18-00283-s001.zip › pharmaceutics-4113934-supplementary.pdf]

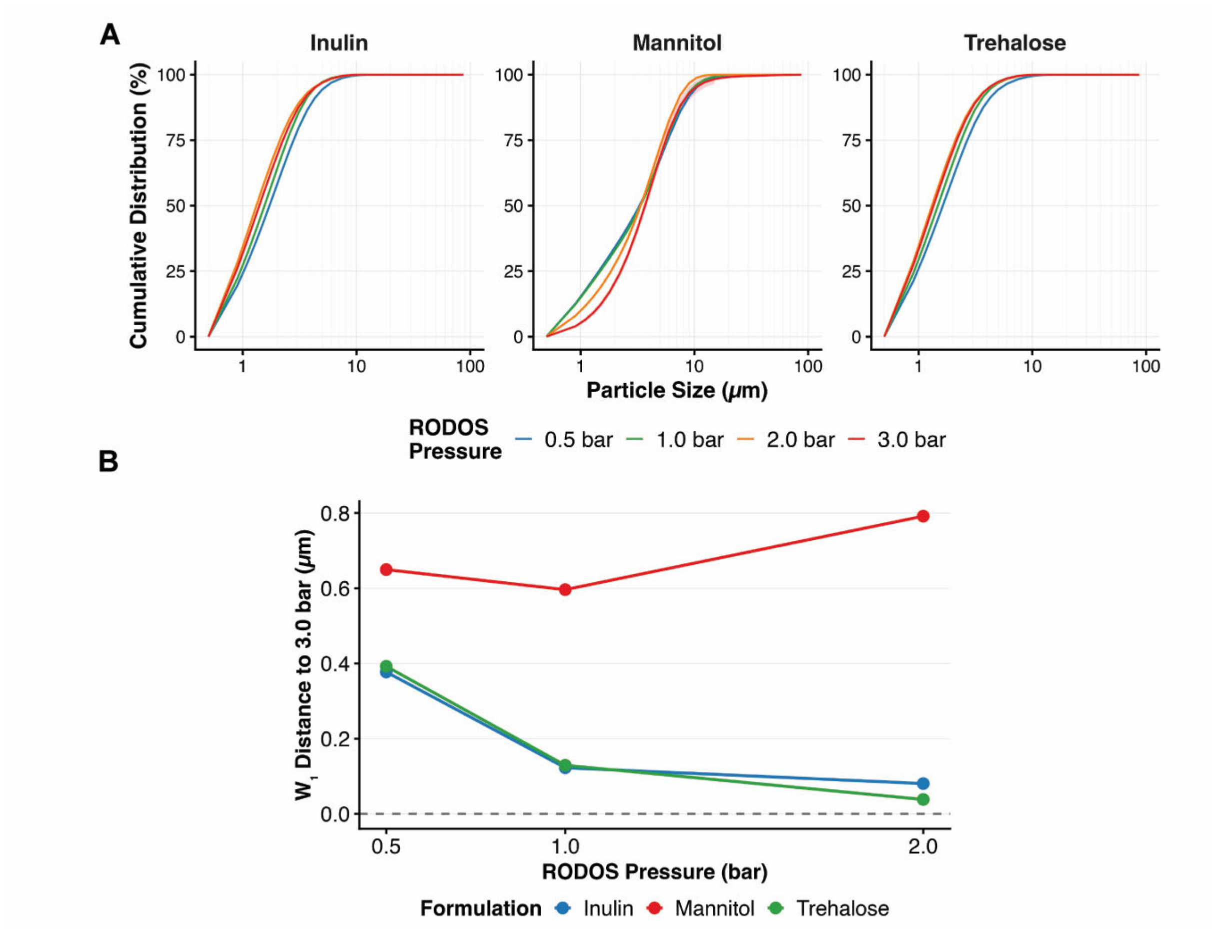

Figure S1. Determination of the maximally dispersed reference state by dry dispersion energy titration using the RODOS module. (A) Cumulative volume-based particle size distributions (Q_3) for spray-dried inulin, mannitol, and trehalose measured by laser diffraction using the RODOS dry dispersion module at increasing dispersion pressures (0.5, 1.0, 2.0, and 3.0 bar). For each formulation, increasing dispersion pressure produces progressively finer distributions that approach a limiting ("plateau") shape, consistent with diminishing changes in the measured PSD at higher dispersion energies. (B) Wasserstein-1 distance (W_1) between particle size distributions measured at lower dispersion pressures (0.5–2.0 bar) and the 3.0 bar condition. For trehalose and inulin, W_1 decreases with increasing dispersion pressure, indicating convergence toward the 3.0 bar distribution. Mannitol exhibits non-monotonic variation across intermediate pressures, consistent with limited redistribution in the measured PSD over this range. However, differences relative to the 3.0 bar condition remain small at 2.0 bar. Collectively, these results support selecting 3.0 bar to represent the reference dispersion energy, evaluated as a practical, distributionally converged reference condition for subsequent analyses.

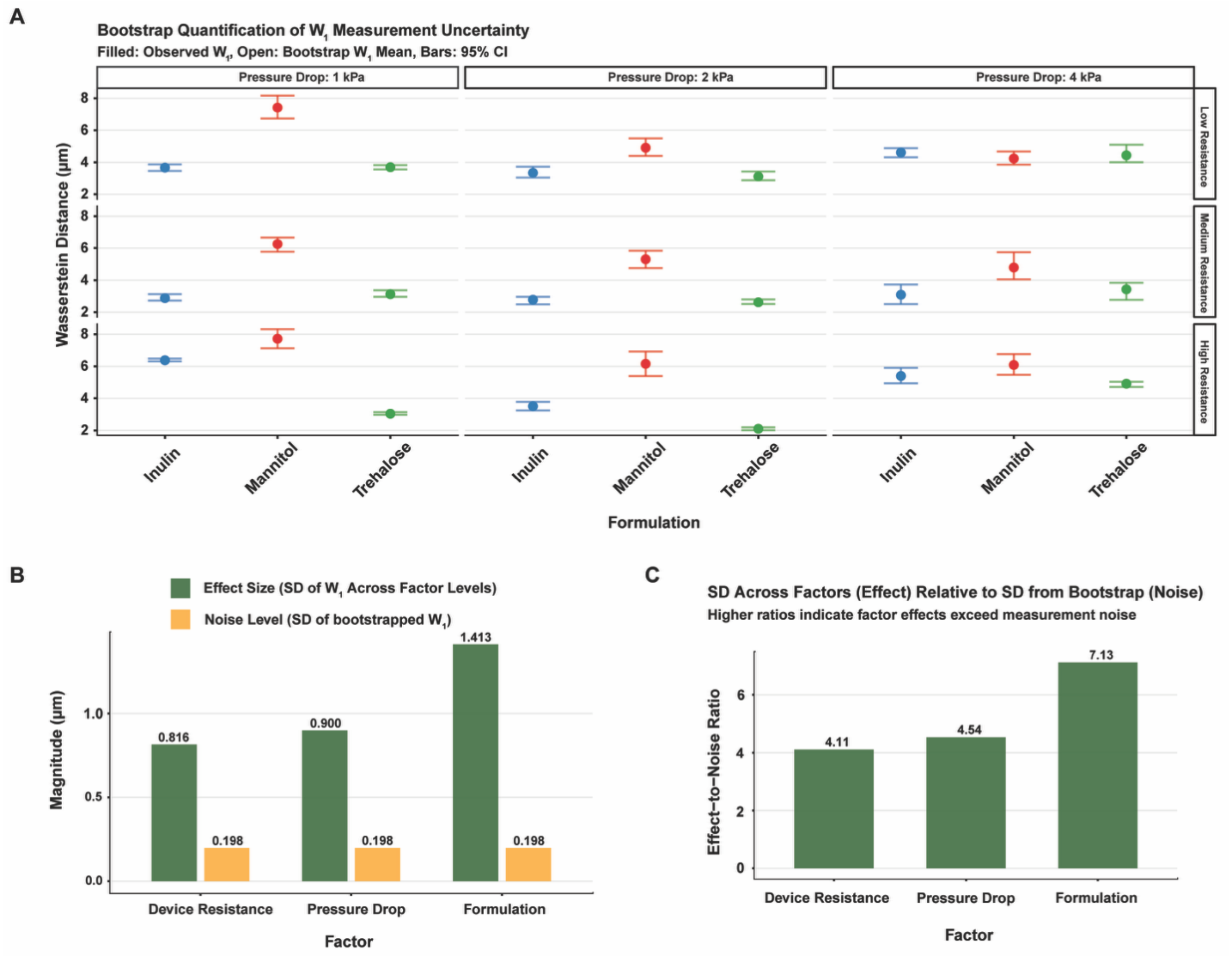

Figure S2. Bootstrap-based analysis of measurement uncertainty and effect magnitude for Wasserstein-1 (W_1) distances. (A) Comparison of experimentally measured W_1 values (filled circles) with bootstrap-derived mean W_1 values (open circles with error bars) across all formulation–device resistance–pressure drop combinations tested. Error bars represent the 95% confidence interval of the bootstrap-derived mean W_1, obtained by resampling replicate laser diffraction measurements with replacement. Across all conditions, the bootstrap-derived mean W_1 overlaps with the corresponding measured W_1 value, indicating minimal within-condition variability in W_1 arising from replicate laser diffraction measurements and demonstrating that measurement noise is small relative to the observed W_1 magnitudes across the design space. (B) W_1 measurement uncertainty and effect magnitudes across device resistance, pressure drop and formulation factors. Effect magnitudes were quantified within each fixed combination of the remaining factors using the across-level range (max − min) of bootstrap-mean W1 values, then averaged across combinations. Noise levels were defined as the mean bootstrap standard deviation within the same combinations. (C) Effect-to-Noise ratios of effect magnitude and noise level across factors (device, resistance, pressure drop, formulation) show that all three experimental factors varied in this study produce W_1 changes several-fold larger than intrinsic measurement uncertainty, demonstrating the separability of factor-driven differences relative to W_1 measurement variability.

Table S1. Comprehensive summary of material properties, morphology, particle size distributions, dispersibility, and aerodynamic performance for spray-dried formulations.

Formulation	Solid State	BET-SSA (m^2/g)	Morphology	RODOS X_{50} ± SD (n = 3; μm)[1]	INHALER X_{50} ± SD (μm)[2]	W_1 (μm)[2]	MMAD ± SD (n = 3; μm)[2]	$FPF_{<5\ \mu m}$ ± SD (n = 3; %)[2]	EF ± SD (n = 3; %)[2]
SD-Trehalose	Amorphous	6.01	Spherical, smooth surfaces	1.36 ± 0.01	3.11 ± 0.09	2.44	2.68 ± 0.19	72.4 ± 3.8	90.2 ± 2.1
SD-Inulin	Amorphous	6.99	Spherical, corrugated surfaces	1.40 ± 0.02	3.35 ± 0.13	2.64	2.64 ± 0.06	74.9 ± 1.4	82.5 ± 3.6
SD-Mannitol	Crystalline	2.19	Angular, faceted morphologies	3.69 ± 0.14	7.73 ± 0.30	4.88	6.36 ± 0.33	24.7 ± 4.2	96.8 ± 1.5

[1]RODOS X_{50} represents the median particle size of the maximally dispersed reference state obtained at 3.0 bar dispersion pressure, while Inhaler X_{50} represents the median particle size of the device-emitted aerosol.
[2]Measurements obtained using medium-resistance RS01 DPI operated at a pressure drop of 2 kPa (n = 3).
W_1 quantifies the Wasserstein-1 distance between inhaler-generated aerosol and RODOS reference distributions.
MMAD = mass median aerodynamic diameter; FPF = fine particle fraction; EF = emitted fraction.
